# Items

**Published:** 1882-12

**Authors:** 


### Items.

MEDICAL NEWS.

THE Fifteenth edition of the United States Dispensatory will be ready in January, 1883. The editors are Dr. H. B. Wood, Professor of Materia Medica and Therapeutics in the University of Pennsylvania, Joseph P. Remington, Professor of Pharmacy, and Samuel B. Sadtler, Professor of Chemistry, in the College of Pharmacy, Philadelphia. The revision has occupied about three years, and has been in all respects most thorough and complete—embracing the most recent discoveries in materia medica, pharmacy, chemistry and therapeutics.

The relation of the work to the United States Pharmacopœia will be fully maintained, whilst the encyclopœdic character of the Dispensatory will be developed to the greatest extent. The new pharmacopœia will be in all its parts fully expounded and discussed, and the most recent non-officinal medicines, as well as those long out of date, will be carefully considered in the second part of the work.

THE Congress of Austrian Archæologists, recently in session at Salzburg, was the scene of an interesting discussion of the human jaw-bone, in which the proportions of a giant were found associated with the teeth of a child, which was dug out, at Stamberg, in Moravia, from under a formation containing bones of the reindeer, snow-owl, cave-bear, and other Arctic animals.—*Popular Science Monthly.*

POPULARITY OF HYPODERMIC SYRINGES.—"There are one hundred hypodermic syringes sold now for each one that found a purchaser a few years ago," said a dealer in surgical instruments, to a New York *Sun* reporter. "People have discovered that they are not only of great service in the alleviation of extreme pain, but that they afford a convenient sort of respectable intoxication."

SINCE the massacre in Alexandria, Egypt has been recommended as a health resort. Dr. Edith Pechey thinks that phthisical and rheumatic patients will be greatly benefited, if they are careful in guarding themselves against the night and morning chills, in taking trips on the Nile.

AT the end of 1881 there were 2,218 registered insane persons in New South Wales, or 119 more than the previous year, and 36 in excess of the average yearly increase. The percentage of deaths for the year was 5.46, the lowest since 1865.—*Austral. Med. Gazette.*

HOMŒOPATHY IN THE COOK COUNTY HOSPITAL, CHICAGO.—Under recent rules, one-fourth of all patients on the medical side, and one-fifth of the surgical cases are placed under the care of the homœopathic internes. No consultations allowed.—*Medical Record.*

MARRIAGE LAWS.—Among the Omahas and Poncas, a man can marry his brother's widow. Widows, however, must wait four years before re-marrying. But, if a widower remains single for two, three, or four years, he must remain so forever.

SOME enterprising dentists, among them Drs. Brophy and Talbot, are about starting in Chicago a Dental Infirmary, for the purpose of giving instruction to medical students, and of furnishing gratuitously to worthy poor their skillful services.

DR. T. DAVIS FITCH has much improved in health. He is now attending to his practice, as before his apoplexy, and conducts successfully his gynæcological clinic at the Woman's Medical College every Saturday afternoon.

AT Philadelphia, October 19, several explosions of sewer gas took place along Twentieth street, and the gas caught on fire. This may suggest either a new mode of constructing sewers or a new means for disinfecting them.

DR. HENRY T. BYFORD has safely returned from his bridal tour, and is again in active pursuit of his professional work. We congratulate him on his marriage, and hope he will add to the number of his illustrious name.

DR. JOHN D. M. CARR, whose death took place recently, has been for fifteen years a well-known physician of this city. In addition to being an excellent physician, he was an active and prominent Free Mason.

THE late epidemic of cholera in Japan caused 2,000 deaths in Yokohama alone. In Manilla, Philippine Islands, the mortality from the same cause numbered from 50 to 300 a day.—*Med. News.*

MR. W. H. RIDEING, in *Harper's Monthly* says that no occupation in the United States stands higher numerically, socially, or financially, than the medical profession.

A CASE of rupture of the stomach is reported in which a dose of lobelia was given to a patient of intemperate habit, by an irregular practitioner. The patient died without vomiting.

ALLUDING to the late troubles in the Cook County Hospital, the *Medical Record* says: "Between politics and homœopathy our Chicago brethren seem to have a hard time.

M. TARNIER of the Maternité, Paris, is the inventor of a baby-incubator which will do for the human race what the egg-incubator has done already for poultry.

MR. T. R. BAKER, in a paper "On the Permeability of the Linings of House Walls to Air," deprecates wall-paper and advocates the old-time whitewashed walls.

Dr. D. G. BRINTON, of Philadelphia, is publishing "The Chronicles of the Mayas," being the first volume of a "Library of Aboriginal American Literature."

THE celebrated author of the Treatise on Comparative Embryology, Prof. F. M. Balfour, lost his life July 19, while ascending the Alps, near Mt. Blanc.

SCARLET fever is widely disseminated through Chicago.

THE Chicago Dental Infirmary has just been licensed by the Secretary of State.

ONE hundred thousand dollars have been appropriated by Congress for the erection of an army and navy hospital at Hot Springs, Arkansas.

SAYS Blackmore: "The common lay mind hates the lawyer, as the lay body objects to the doctor, and the soul is timorous of the parson."

THE annual congress of the German Anthropological Society met at Frankfort, August 14, with five hundred members in attendance.

THE celebrated chemist, Friederich Woehler, Director of the Chemical Institute at Göttingen, died September 25, aged 82 years.

ANTHROPOLOGY.—What seems evident foot-prints of men have been found at Carson City, Nevada, in a Quarternary sandstone.

Two hundred and sixty-seven medical works were published in Japan in the Japanese language in 1881.—*Med. News.*

THE State of Maine has recently elected Dr. Frederick Robie, Governor.

